# Longitudinal active sampling for respiratory viral infections across age groups

**DOI:** 10.1111/irv.12629

**Published:** 2019-02-15

**Authors:** Marta Galanti, Ruthie Birger, Minhaz Ud‐Dean, Ioan Filip, Haruka Morita, Devon Comito, Simon Anthony, Greg A. Freyer, Sadiat Ibrahim, Benjamin Lane, Chanel Ligon, Raul Rabadan, Atinuke Shittu, Eudosie Tagne, Jeffrey Shaman

**Affiliations:** ^1^ Department of Environmental Health Sciences Mailman School of Public Health Columbia University New York New York; ^2^ Department of Systems Biology Columbia University New York New York; ^3^ Department of Epidemiology Columbia University New York New York

**Keywords:** prevalence of respiratory viruses, respiratory viral infections, seasonality of respiratory viruses, susceptibility to respiratory infections

## Abstract

**Background:**

Respiratory viral infections are a major cause of morbidity and mortality worldwide. However, their characterization is incomplete because prevalence estimates are based on syndromic surveillance data. Here, we address this shortcoming through the analysis of infection rates among individuals tested regularly for respiratory viral infections, irrespective of their symptoms.

**Methods:**

We carried out longitudinal sampling and analysis among 214 individuals enrolled at multiple New York City locations from fall 2016 to spring 2018. We combined personal information with weekly nasal swab collection to investigate the prevalence of 18 respiratory viruses among different age groups and to assess risk factors associated with infection susceptibility.

**Results:**

17.5% of samples were positive for respiratory viruses. Some viruses circulated predominantly during winter, whereas others were found year round. Rhinovirus and coronavirus were most frequently detected. Children registered the highest positivity rates, and adults with daily contacts with children experienced significantly more infections than their counterparts without children.

**Conclusion:**

Respiratory viral infections are widespread among the general population with the majority of individuals presenting multiple infections per year. The observations identify children as the principal source of respiratory infections. These findings motivate further active surveillance and analysis of differences in pathogenicity among respiratory viruses.

## 1 INTRODUCTION

Traditional estimates of influenza‐like infection rates are based on specimens collected from patients seeking treatment in medical clinics or emergency departments.^1^, ^2^ This surveillance overlooks the large part of the population who experience asymptomatic infections or choose not to see a doctor. As a result, prevalence estimates of different viruses are skewed toward the most pathogenic agents, and overall infection rates are likely underestimated. Here, we supplement traditional surveillance through sampling among healthy individuals who are tested weekly for multiple respiratory viruses irrespective of whether they are experiencing respiratory symptoms. We use this information to quantify the prevalence of common respiratory viruses within the general population, characterize the seasonality of these viruses, and assess the risk factors (age, habits, and health conditions) that increase susceptibility among hosts.

## 2 METHODS

### 2.1 Cohort composition and survey

We enrolled 214 healthy individuals from different locations in the borough of Manhattan in New York City. The cohort included children attending 2 day cares, along with their siblings and their parents, teenagers and teachers from a high school, adults working at two emergency departments (a pediatric ER and an adult ER), and adults working at a university medical center. The study period spanned two years between October 2016 and April 2018 with some individuals enrolled for a single cold and flu season (October‐April) and others for the entire period. At enrollment, individuals were asked to complete a baseline survey and provide two nasopharyngeal swab samples (one for each nostril). Following this preliminary step, two nasopharyngeal samples were collected weekly from each participant irrespective of symptoms. The baseline questionnaire completed at the time of enrollment included information on ethnicity, general health, daily habits, travel history, and household structure. Parents provided consent and questionnaire answers for children enrolled. Details on the participants are summarized in Table 1.

**Table 1:** Demographics of the study cohort

	Children	Parents	Teenagers	Teachers	Peds ED	Adult ED	Medical Center	All Cohorts
	n %	n %	n %	n %	n %	n %	n %	n %
Enrolled	35 (16.4)	20 (9.3)	42 (19.6)	15 (7.0)	22 (10.3)	11 (5.2)	69 (32.2)	214 (100)
Total samples	1016 (24.1)	524(12.4)	361 (8.6)	248 (5.9)	537 (12.7)	103 (2.4)	1426 (33.8)	4215 (100)
Years (% per cohort)								
One	8 (22.9)	8 (40.0)	33 (78.6)	7 (46.6)	11 (50.0)	0 (0)	15 (21.7)	82 (38.3)
Two	10 (28.6)	6 (30.0)	9 (21.4)	1 (6.7)	2 (9.1)	11 (100)	41 (59.4)	80 (37.4)
Both	17 (48.5)	6 (30.0)	0 (0)	7 (46.6)	9 (40.9)	0 (0)	13 (18.9)	52 (24.3)
Gender (% per cohort)								
Male	17 (48.6)	3 (15.0)	27 (64.3)	8 (53.3)	7 (31.8)	4 (36.4)	27(39.1)	93 (43.5)
Female	18 (51.4)	17 (85.0)	15 (35.7)	7 (46.7)	15 (68.2)	7 (63.6)	41 (59.4)	120 (56.0)
Transgender	0 (0)	0 (0)	0 (0)	0 (0)	0 (0)	0 (0)	1 (1.5)	1 (0.5)
Age								
Range	0‐9	24‐43	14‐18	24‐38	24‐61	25‐63	20‐63	0‐63
Median	3	33	14	27	39	33.5	26	25
Hispanic (% per cohort)								
Yes	25 (71.4)	8 (40.0)	20 (47.6)	2 (13.3)	1 (4.5)	0 (0)	12 (17.4)	68 (31.8)
No	10 (28.6)	10 (50.0)	21 (50.0)	13 (86.7)	21(95.5)	11 (100)	57 (82.6)	143 (66.8)
Don't know	0 (0)	2 (10.10)	1 (2.4)	0 (0)	0 (0)	0 (0)	0 (0)	3 (1.4)
Race (% per cohort)								
White	3 (8.6)	4 (20.0)	1 (2.4)	9 (60.0)	18 (81.8)	5 (45.5)	37 (53.6)	77 (36.0)
African American	3 (8.6)	2 (10.0)	21 (50.0)	4 (26.7)	0 (0)	2 (18.2)	4 (5.8)	36 (16.8)
Asian	3 (8.6)	3 (15.0)	2 (4.8)	1 (6.7)	3 (13.6)	3 (27.3)	21 (30.4)	36 (16.8)
American Indian	19 (54.2)	5 (25.0)	1 (2.4)	0 (0)	0 (0)	0 (0)	1 (1.5)	26 (12.1)
Other Pacific	0 (0)	0 (0)	1 (2.4)	0 (0)	0 (0)	0 (0)	0 (0)	1 (0.5)
Other or mixed	7 (20.0)	6 (30.0)	10 (23.8)	1 (6.7)	0 (0)	1 (9.0)	5 (7.2)	30 (14.0)
Don't know	0 (0)	0 (0)	6 (14.3)	0 (0)	1 (4.6)	0 (0)	1 (1.5)	8 (3.7)

### 2.2 Specimen collection and analysis

Two nasopharyngeal samples per participant were collected on a weekly basis using minitip flocked swabs. Both samples were stored jointly in 2 mL DNA/RNA Shield (Zymo Research, Irvine, CA) at 4‐25°C for up to 30 days and then stored at −80°C in two aliquots. Nucleic acids were extracted from 200 uL of sample and 10 uL of internal control using the EasyMAG NucliSENS system (bioMerieux, Durham, NC). Samples were then screened for viruses using the eSensor XT‐8 respiratory viral panel (RVP; GenMark Dx, Carlsbad, CA),^3^ a multiplex PCR assay. The RVP system separately detects influenza A (any subtype, A/H1N1, A/H3N2, A/H1N1pdm2009) and B; RSV A and B; parainfluenza (PIV) 1, 2, 3, and 4; human metapneumovirus (HMPV); human rhinovirus (HRV); adenovirus B/E and C; and coronaviruses (CoV) 229E, NL63, OC43, and HKU1. Samples positive for a particular virus were identified by an electrical signal intensity of ≥2 nA/mm^2^ (with the exception of Coronavirus OC43 for which positive results were identified by an intensity of ≥25 nA, per manufacturer specifications).

### 2.3 Statistical analysis

Analyses were conducted using the total number of positive samples, as well as the number of illness events. We defined an illness event as a group of consecutive weekly swab specimens for a given individual that were positive for the same virus (allowing for a 1‐week gap to account for false negatives and temporary low shedding).

The impact of population‐based variables on virus positivity or illness event rates was assessed using ANOVA and logistic regression. The chi‐squared statistic was also used to assess pairwise differences. The participants were divided into four groups as follows: children (under 10 years of age), teenagers, adults with daily contact with children (parents and PEDS ER doctors), and adults without daily contact with children. For this analysis, only the 192 participants who contributed at least six separate pairs of nasopharyngeal samples were included.

## 3 RESULTS

A total of 4215 samples were collected and analyzed. Among them, 737 (17.5%) tested positive for one or more of the respiratory viruses. Seventy‐two samples tested positive for multiple viruses (10% of the positive tests). Across time, between 10% and 25% of the samples tested positive each week, this overall rate of positivity did not exhibit a trend or seasonality.

**Figure 1:**
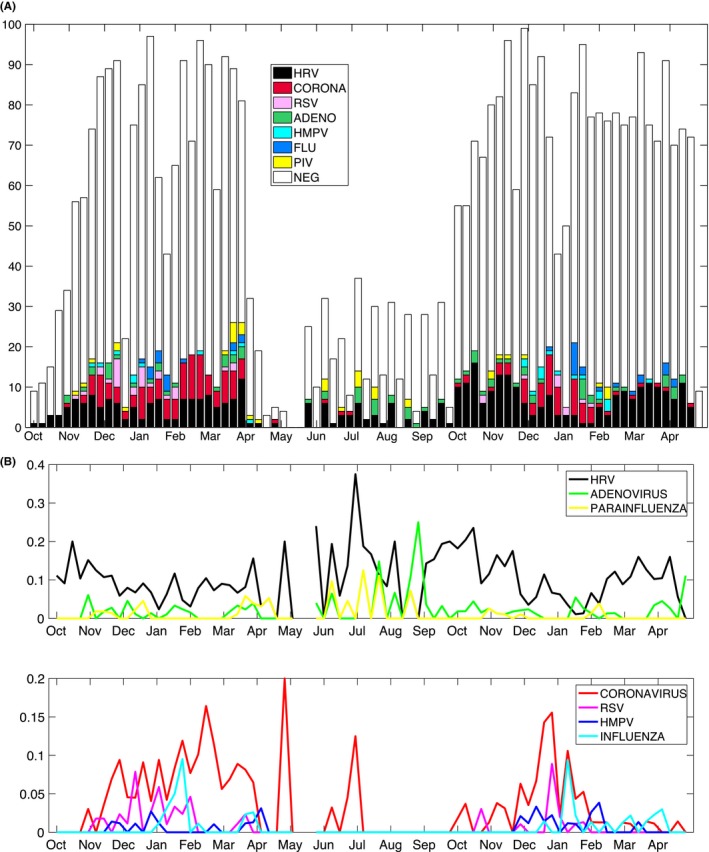
A, Positive (color) and negative (white bars) samples aggregated by week. The weekly distribution for each age group is shown in the Supporting information. B, Time series highlighting the distinction between viruses with and without a seasonal pattern. Weekly positive results for each virus are normalized on the total number of tests collected within the week. Fewer samples were collected in late spring and summer. The spikes in prevalence of coronavirus in May and July are likely artifacts due to the lower numbers of samples collected

Rhinovirus and coronavirus were the most frequently identified viruses, present in 408 and 188 samples (55% and 25% of the positive samples), respectively, followed by adenovirus (11%), RSV (5%), influenza (5%), parainfluenza virus (4%), and HMPV (3%). Among these viruses, influenza, RSV, coronavirus, and HMPV were most prevalent in the winter months and had no documented incidence during the summer months. In contrast, rhinovirus, adenovirus, and parainfluenza circulated throughout the entire study period (the temporal distribution is showed in Figure 1 and Supplementary Figure S1).

We compared the results among the following four cohort groups: children, teenagers, adults with daily contact with children, and adults without daily contact with children. Children presented a significantly higher number of co‐infections than the other groups: 16% of positive results among children were positive for more than one virus vs 0%, 2%, and 6%, respectively, for teenagers, adults without children, and adults with children (significantly higher for the children, *P *<* *0.0001).

**Figure 2:**
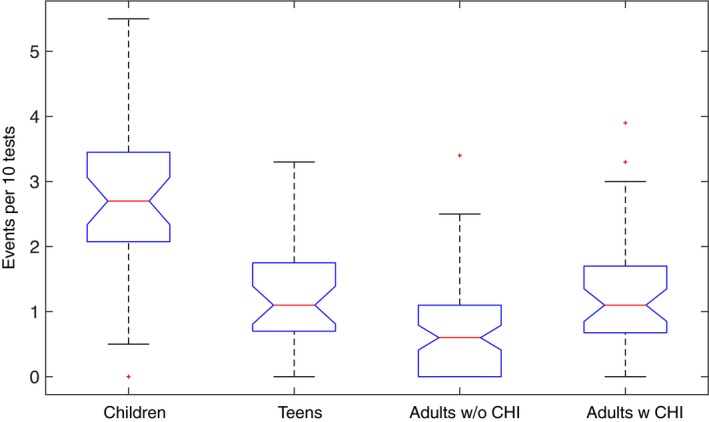
Distribution of the number of infection events per 10 tests among the four groups. The boxplots show the difference among the medians of the groups (red lines). Where the notches in the boxplot do not overlap, it is possible to state at 95% confidence level that the true medians differ

The percentage of tests that were positive differed significantly among the groups: 36% for children, 15% for teenagers, 17% for the adults with children, and only 7% for adults without children. The odds of testing positive for children were six times higher than the odds of testing positive for adults without daily contacts with children (see Supplementary Figure S2 for raw numbers across the different locations and Table S1 for results of logistic regression). The analysis of viral events also confirmed a significant difference across groups, with children exhibiting the highest number of viral events and adults without children the lowest. Comparison of the number of viral events among the four groups is shown in Figure 2, together with *P*‐values for pairwise comparisons (Table 2).

**Table 2:** The table shows the pairwise differences among the means of each group along with 95% confidence intervals and *P*‐values

Group 1	Group 2	Diff. Means (95% CI of difference)	*P* Value
Children	Teens	1.43 (0.85, 2.00)	5.13 × 10^−9^
Children	Adults w/o chi	1.99 (1.52, 2.48)	3.77 × 10^−9^
Children	Adults w chi	1.41 (0.87, 1.96)	3.91 × 10^−9^
Teens	Adults w/o chi	0.57 (0.08, 1.07)	0.01
Teens	Adults w chi	−0.01 (−0.57, 0.54)	0.99
Adults w/o chi	Adults w chi	−0.59 (−1.04, −0.13)	0.005

We tested the effect of several baseline factors on the normalized number of infections. Gender, presence of pre‐existing respiratory conditions (any condition, but also separately asthma and allergy), choice of public vs private means of transportation, and self‐identification with *Hispanic* ethnicity did not have a significant association with the number of infections. In contrast, age group, living with other children, and self‐identification with *American Indian* race had a significant effect on the number of infections per 10 test (*P*‐values respectively 0, 0.05, and 0.01). Note that the majority (73%) of people self‐identifying as American Indian were children.

**Figure 3:**
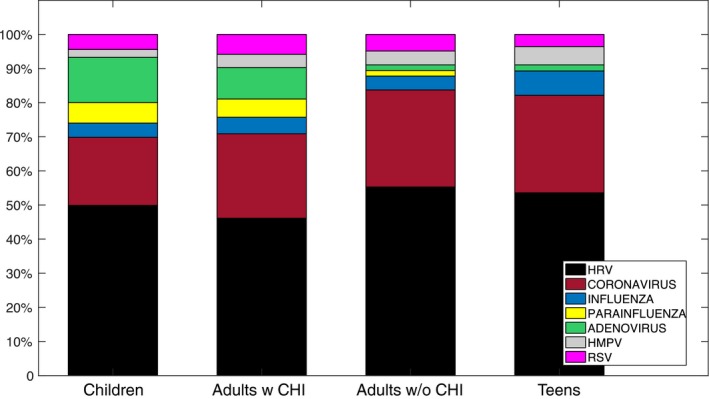
Percentage of respiratory virus infections due to each agent across the four groups

The distribution of viruses found across the different age groups was similar, with coronavirus and rhinovirus accounting for 70% to 82% of positive results. Children and adults with daily contacts with children had similar distributions of infecting pathogens, with higher percentages of adenovirus and parainfluenza than the other groups (Figure 3). Multiple subsequent infections with the same virus were frequently identified for HRV and coronavirus in all groups and for adenovirus among the children. In particular, multiple infections with rhinovirus were documented for 30% of the participants, with a maximum of 7 separate HRV events within a year.

## 4 DISCUSSION

Respiratory viruses are the most common cause of infection among the general population. Every year, they cause millions of illnesses, thousands of hospitalizations and deaths,^4^ and impose a significant economic burden on health care systems.^5^ Respiratory viral infections are highly contagious, and the lack of a vaccine (with the exception of influenza) and absence of long‐lasting immunity favors the continued recurrence of outbreaks of these pathogens. Despite this ubiquitous circulation, very little is known about the prevalence of these infections among the global population, as the available estimates are based on syndromic surveillance (eg, in the United States, the CDC‐based National Respiratory and Enteric Virus Surveillance System (NREVSS) and FLUVIEW^1^, ^2^) and prospective studies are usually restricted to groups at risk.

Most of the existing literature focuses on young children because acute respiratory infections are a leading cause of childhood hospitalization and mortality worldwide.^6^, ^7^ Early life respiratory viral infections, principally due to rhinovirus and RSV, have been shown to be associated with the development of recurrent wheezing and asthma in infants and children.^8^, ^9^, ^10^ Conversely, the impact of these infectious agents on healthy adults and the role of asymptomatic infections on transmission dynamics have not been properly investigated. A recent study found high levels of asymptomatic respiratory infection among an ambulatory adult population in New York City.^11^

The work presented here is part of larger project attempting to document the prevalence of respiratory viral infections among different strata of the population, with a specific focus on the environmental, demographic, and genetic factors affecting susceptibility, symptomology and transmission. Here, we have shown that respiratory virus infections are present among all age groups, with almost all participants presenting with at least one viral infection per year and an overall rate of 17.5% of positivity among all collected samples. Infection appeared to be strongly connected with age, with young children presenting more than double the number of infections of other age groups. Adults with daily contact with children (parents and pediatric doctors) also had a higher number of infections than their counterparts without daily contact with children. Moreover, the distribution of respiratory virus infections for parents and pediatric doctors was very similar to the distribution observed in the children. These observations suggest children are a principal source of respiratory infection and confirm earlier studies that found day cares to be optimal environments for transmission.^12^, ^13^ Self‐identification with American Indian or Alaskan native race was also a factor influencing the number of respiratory viral infections. This association was likely due to the non‐mixed nature of our population, as nearly all of the participants self‐identifying as Alaskan native or American Indian were children or parents from one of the day care settings. Children were also associated with a higher risk for co‐infection than adults and teenagers, as has also been shown in earlier studies.^14^

A larger variety of viruses was found in children and their close contacts; however, rhinovirus and coronaviruses were the most frequently identified viral respiratory pathogens in all age groups. Together, these two viruses accounted for more than 70% of positive results. The presence of multiple subsequent infections with the same virus in many individuals suggests short‐lasting immunity or potential low cross‐immunity among multiple co‐circulating serotypes of the same pathogen. Previous studies on HRV report up to 20 different rhinovirus types (among more than one hundred known) circulating in a community during one season. Further, the prevailing strains can differ widely between locations, across seasons, and switch almost completely from year to year.^15^

Our estimates of incidence rates differ markedly from those built on syndromic surveillance data.^16^, ^17^ Among patients seeking care, some viruses like influenza are overrepresented and others, like coronaviruses, profoundly underrepresented. This asymmetry is likely due to the different pathogenicity of the viruses causing respiratory infections and underscores the importance of using of non‐syndromic surveillance data to capture the true overall prevalence of respiratory virus infection within the general population.

A limitation of this study is the low frequency of sampling in late spring/summer months, due to decreased participation of the enrolled individuals. Despite the lower number of samples collected during these months, seasonal and non‐seasonal patterns are clearly identifiable. Some viruses (influenza, RSV, coronavirus and HMPV) had a distinct peak during winter months, whereas others circulated year round. Such assessment of seasonality for different pathogens is important for planning vaccination and control strategies and to understand the dynamics of transmission.

Future work should involve analyses of differences in pathogenicity among respiratory viruses, as well as the impact of genetic, demographic, and environmental features on pathogenicity. Moreover, longitudinal sampling coupled with information on symptomology should be used to analyze the impact of asymptomatic infections and the role of asymptomatic carriers on transmission dynamics.

## References

[1] CDC . NREVVS. Available at: https://www.cdc.gov/surveillance/nrevss/index.html , accessed October 2018.

[2] CDC . FLUVIEW. Available at: https://www.cdc.gov/flu/weekly/index.htm , accessed October 2018.

[3] Popowitch EB , O'Neill SS , Miller MB . Comparison of the Biofire FilmArray RP, Genmark eSensor RVP, Luminex xTAG RVPv1, and Luminex xTAG RVP Fast Multiplex Assays for Detection of Respiratory Viruses. J Clin Microbiol. 2013;51:1528‐1533. 23486707 10.1128/JCM.03368-12 PMC3647947

[4] Thompson WW , Shay DK , Weintraub E , et al. Mortality associated with influenza and respiratory syncytial virus in the united states. JAMA. 2003;289(2):179‐186. 12517228 10.1001/jama.289.2.179

[5] Fendrick A , Monto AS , Nightengale B , Sarnes M . The economic burden of non–influenza‐related viral respiratory tract infection in the united states. Arch Intern Med. 2003;163(4):487‐494. 12588210 10.1001/archinte.163.4.487

[6] Nair H , Nokes DJ , Gessner BD , et al. Global burden of acute lower respiratory infections due to respiratory syncytial virus in young children: a systematic review and meta‐analysis. Lancet. 2010;375(9725):1545‐1555. 20399493 10.1016/S0140-6736(10)60206-1 PMC2864404

[7] Berman S . Epidemiology of acute respiratory infections in children of developing countries. Rev Infect Dis. 1991;13:S454‐S462. 1862276 10.1093/clinids/13.supplement_6.s454

[8] Kusel MMH , de Klerk NH , Kebadze T , et al. Early‐life respiratory viral infections, atopic sensitization, and risk of subsequent development of persistent asthma. J Allergy Clin Immunol. 2007;119(5):1105‐1110. 17353039 10.1016/j.jaci.2006.12.669 PMC7125611

[9] Busse WW , Lemanske RF , Gern JE . Role of viral respiratory infections in asthma and asthma exacerbations. Lancet Infect Dis. 2010;376:826‐834. 10.1016/S0140-6736(10)61380-3 PMC2972660 20816549

[10] Jackson DJ , Gangnon RE , Evans MD , et al. Wheezing Rhinovirus Illnesses in Early Life Predict Asthma Development in High‐Risk Children. Am J Respir Crit Care Med. 2008;178(7):667‐672. 18565953 10.1164/rccm.200802-309OC PMC2556448

[11] Birger R , Morita H , Comito D , et al. Asymptomatic Shedding of Respiratory Virus among an Ambulatory Population across Seasons. mSphere, 2018;3(4):e00249‐18. 29997120 10.1128/mSphere.00249-18 PMC6041500

[12] Schwartz B , Giebink GS , Henderson FW , Reichler MR , Jereb J , Collet JP . Respiratory infections in day care. Pediatrics. 1994;94:1018‐1020. 7971043

[13] Wald ER , Dashefsky B , Byers C , Guerra N , Taylor F . Frequency and severity of infections in day care. J Pediatr. 1988;112(4):540‐546. 3351677 10.1016/s0022-3476(88)80164-1

[14] Scotta MC , Chakr VCBG , de Moura A , et al. Respiratory viral coinfection and disease severity in children: a systematic review and meta‐analysis. J Clin Virol. 2016;80:45‐56. 27155055 10.1016/j.jcv.2016.04.019 PMC7185664

[15] Gavala M , Bertics PJ , Gern JE . Rhinoviruses, Allergic Inflammation, and Asthma. Immunol Rev. 2011;242(1):69‐90. 21682739 10.1111/j.1600-065X.2011.01031.x PMC3119863

[16] Visseaux B , Burdet C , Voiriot G , et al. Prevalence of respiratory viruses among adults, by season, age, respiratory tract region and type of medical unit in Paris, France, from 2011 to 2016. PLoS ONE. 2017;12(7):e0180888. 28708843 10.1371/journal.pone.0180888 PMC5510824

[17] Tregoning JS , Schwarze J . Respiratory Viral Infections in Infants: causes, Clinical Symptoms, Virology, and Immunology. Clin Microbiol Rev. 2010;23(1):74‐98. 20065326 10.1128/CMR.00032-09 PMC2806659

