# Supplementary material for: Longitudinal active sampling for respiratory viral infections across age groups
Source: Influenza Other Respir Viruses. 2019 Feb 15;13(3):226–32. doi: 10.1111/irv.12629 (PMC6468062; doi:10.1111/irv.12629)
Supplement: Supplementary file 1 [file IRV-13-226-s001.docx]

**Fig S1**: Temporal distribution of viral infections across the different groups
