# Supplementary material for: Longitudinal active sampling for respiratory viral infections across age groups
Source: Influenza Other Respir Viruses. 2019 Feb 15;13(3):226–32. doi: 10.1111/irv.12629 (PMC6468062; doi:10.1111/irv.12629)
Supplement: Supplementary file 3 [file IRV-13-226-s003.docx]

**Table S1:** Logistic regression model describing the odds of testing positive for one (or more) respiratory virus among age groups. The baseline group is the group of adults without daily contacts with children.

|  | Coefficient | Std. Err. | p value | Odds Ratio |
| --- | --- | --- | --- | --- |
| Intercept | -2.421 | 0.087 | 0.000 | 0.0889 |
| Teenagers | 0.726 | 0.169 | 0.000 | 2.066 |
| Adults w. Chi | 0.771 | 0.120 | 0.000 | 2.162 |
| Children | 1.84 | 0.109 | 0.000 | 6.310 |
